# Mortality and survival of COVID-19

**DOI:** 10.1017/S0950268820001405

**Published:** 2020-06-25

**Authors:** G. J. B. Sousa, T. S. Garces, V. R. F. Cestari, R. S. Florêncio, T. M. M. Moreira, M. L. D. Pereira

**Affiliations:** Postgraduation Program in Clinical Care in Nursing and Health, Ceará State University, Fortaleza, Ceará, Brazil

**Keywords:** Brazil, COVID-19, epidemiology, mortality, survival

## Abstract

This study aims to identify the risk factors associated with mortality and survival of COVID-19 cases in a state of the Brazilian Northeast. It is a historical cohort with a secondary database of 2070 people that presented flu-like symptoms, sought health assistance in the state and tested positive to COVID-19 until 14 April 2020, only moderate and severe cases were hospitalised. The main outcome was death as a binary variable (yes/no). It also investigated the main factors related to mortality and survival of the disease. Time since the beginning of symptoms until death/end of the survey (14 April 2020) was the time variable of this study. Mortality was analysed by robust Poisson regression, and survival by Kaplan–Meier and Cox regression. From the 2070 people that tested positive to COVID-19, 131 (6.3%) died and 1939 (93.7%) survived, the overall survival probability was 87.7% from the 24th day of infection. Mortality was enhanced by the variables: elderly (HR 3.6; 95% CI 2.3–5.8; *P* < 0.001), neurological diseases (HR 3.9; 95% CI 1.9–7.8; *P* < 0.001), pneumopathies (HR 2.6; 95% CI 1.4–4.7; *P* < 0.001) and cardiovascular diseases (HR 8.9; 95% CI 5.4–14.5; *P* < 0.001). In conclusion, mortality by COVID-19 in Ceará is similar to countries with a large number of cases of the disease, although deaths occur later. Elderly people and comorbidities presented a greater risk of death.

## Introduction

National and international health agencies have been monitoring the mortality of the new coronavirus day after day. This has so far ranged from 0.9% in Russia to 18.3% in France. What determines such variation is that in some countries the epidemic has not yet reached its peak, there is a lot of underreporting and the case definition itself is not standardised [1–3].

In Brazil, the first confirmed case of coronavirus disease 2019 (COVID-19) took place in São Paulo in late February 2020, and on 20 March, community transmission of the disease was announced (BRASIL, 2020). Currently, there are more than 60 000 cases and almost 5000 deaths by COVID-19 in the country, although its incidence and mortality curve is still rising. Brazil is the 11th country in the number of cases (52 995) in the world and has a high mortality rate (6.9%), which is higher than the USA, the country in the globe with the highest number of cases. In this scenario, São Paulo has the highest number of Brazilian cases (17 826), followed by Rio de Janeiro, which also has the highest mortality rate (9.1%), and Ceará third in the country in the number of cases [3, 4].

Ceará is located in the Northeast of Brazil, which accounts for one-third of all cases reported in the country. Regionally, Ceará is the state with the highest number of confirmed cases of the disease. Its capital, Fortaleza, has 90% of the cases in the state and the highest demographic density among Brazilian capitals, enhancing transmission. On the other hand, Fortaleza is one of the most popular tourist destinations, either for its nature and landscape or for its culture and attractions. It is an important economic centre with recent integration into Europe through an Air Hub, which is thought to have contributed to placing it as the epicentre of COVID-19 in the Northeast and Brazil, as it has the highest transmission rate and the highest incidence of the disease [5]. As a result, despite that the Ceará State Government has created almost 400 hospital beds until 25 April, currently, 84% of the intensive care units (ICU) are filled, 96% of them by patients with severe COVID-19 [5]. Even though efforts are made to face the pandemic, deaths tend to increase over days. Then, it is essential to study which factors influence the risk of death from COVID-19 and how they are influenced by time. Thus, this research aims to identify the risk factors for mortality and survival of COVID-19 cases in the state.

## Methods

Historical cohort was conducted in Fortaleza (Ceará's capital city) in 2020 by using IntegraSUS data (https://integrasus.saude.ce.gov.br/). It is a free-access website that holds data and indicators of COVID-19 in the Ceará State [5].

This system contains all cases of people that presented flu-like syndrome in the state and was tested to COVID-19. It is daily updated regarding new cases, test results and patient's treatment outcomes. Moreover, given the proportion of the disease in Ceará, only people that needed medical assistance and went to healthcare services were tested; from those, moderate and severe cases were hospitalised and received health assistance. The cause of death was determined by the physician given positive laboratory tests to COVID-19 and the clinic manifestation of the disease. However, it is important to highlight that the system is subject to underreporting and then cases and death numbers may be greater than those reported.

All 19 964 cases tested until 14 April 2020 were included in the study and 17 894 were excluded because they tested negative; so 2070 cases that tested positive to COVID-19 remained as this study's sample. Death by COVID-19 (yes/no) was defined as the outcome variable. The selected predictors were age range (elderly/not elderly, once Brazil considers elderly those with ≥60 years) and sex. Clinical variables were asthma, pneumopathies, cardiovascular disease (CVD), diabetes, hematologic diseases, immunodeficiencies, neurological diseases, renal diseases, Down's syndrome and being in the puerperium. Moreover, hospitalisation and ICU admission were included as predictors.

Dates were available in the database and then, the following temporal intervals were created: (T1) time since the beginning of symptoms until ICU admission (in days – d), (T2) time since the beginning of symptoms until death/end of the survey (14 April 2020) (d) and (T3) time since ICU admission until death/end of the survey (d). Due to missing values, T1 and T3 were only descriptively analysed.

Other analyses included simple and relative frequencies, association tests among categorical variables by *χ*^2^ test (*P* < 0.05) and relative risk with 95% confidence intervals (95% CI). Following this, variables with *P* < 0.20 were included in a robust Poisson regression model.

In the survival analysis, the outcome was tested related to T2 (time since the beginning of symptoms until death/end of the survey). For people who did not die, 14 April was included as the final day of research; therefore, the cohort ended. Thus, it was possible to calculate the probability of COVID-19 death from the 1st, 10th and 20th days since the beginning of symptoms. The Kaplan–Meier survival function was used by the log-rank test to determine differences in survival rates, considered different when *P* < 0.05. Finally, as a sensitivity analysis, variables with statistically different survival curves were included in a Cox regression model. All analyses were performed in Stata 12.

The study dismissed previous approval of the Ethics of Research Committee because the database is in the public domain and did not have identification such as name or address. Even though the research did not need approval, the researchers state their ethical commitment in handling, analysing and publishing data according to the Resolution 466/12 by the Brazilian Research Council.

## Results

Two thousand and seventy confirmed cases of COVID-19 were analysed. They presented a median of 44 years (IQR 34–59) and the elderly represent 24% of the total. The disease affected both sexes in almost the same proportion. Regarding the comorbidities, a higher frequency of CVD (7.3%), diabetes (5.5%) and pneumopathies (1.2%) was observed.

Furthermore, 11.4% of the patients were hospitalised and 5.4% were admitted to ICU, and the cumulative incidence of mortality of the disease was 6.3% (Table 1). A median of 19 days since the beginning of symptoms until death/end of the survey (IQR 12–23), a median of 6 days since the beginning of symptoms until ICU admission (IQR 3–9.5) and a median of 12.5 days since ICU admission until death/end of the survey (IQR 8–17) were identified.

**Table 1.** COVID-19 confirmed cases, according to the death event

Variables	Total	Death by COVID-19		*P*-value	RR	95% CI
		Yes	No			
	No. (%)	No. (%)	No. (%)			
Age range				<0.001		
<60 years	1573 (76.0)	32 (2.0)	1541 (98.0)		1	
≥60 years	597 (24.0)	99 (19.9)	398 (80.1)		3.7	3.2–4.2
Sex				0.119		
Male	1017 (49.1)	73 (7.2)	944 (92.8)		1.1	0.9–1.3
Female	1053 (50.9)	58 (5.5)	995 (94.5)		1	
Asthma				0.209		
Yes	5 (0.2)	1 (20.0)	4 (80.0)		3.7	0.4–32.9
No	2065 (99.8)	130 (6.3)	1935 (93.7)		1	
Cardiovascular diseases				<0.001		
Yes	152 (7.3)	86 (56.6)	66 (43.4)		19.3	14.7–25.2
No	1918 (92.7)	45 (2.3)	1837 (97.7)		1	
Diabetes				<0.001		
Yes	114 (5.5)	56 (49.1)	58 (50.9)		14.3	10.3–19.7
No	1956 (94.5)	75 (3.8)	1881 (96.2)		1	
Hematologic diseases				<0.001		
Yes	4 (0.2)	2 (50.0)	2 (50.0)		14.8	2.1–104.2
No	2066 (99.8)	129 (6.2)	1937 (93.8)		1	
Immunodeficiencies				0.652		
Yes	3 (0.1)	0 (0.0)	3 (100.0)		–	
No	2067 (99.9)	131 (6.3)	1936 (93.7)		1	
Neurological diseases				<0.001		
Yes	16 (0.8)	11 (68.7)	5 (31.2)		32.6	11.5–92.3
No	2054 (99.2)	120 (5.8)	1934 (94.2)		1	
Obesity				<0.001		
Yes	4 (0.2)	2 (50.0)	2 (50.0)		14.8	2.1–104.2
No	2065 (99.8)	129 (6.2)	1936 (93.8)		1	
Pneumopathies				<0.001		
Yes	25 (1.2)	13 (52.0)	12 (48.0)		16.0	7.5–34.4
No	2045 (98.8)	118 (5.8)	1927 (94.2)		1	
Puerperium				0.713		
Yes	2 (0.1)	0 (0.0)	2 (100.0)		–	
No	2068 (99.9)	131 (6.3)	1937 (93.7)		1	
Renal diseases				<0.001		
Yes	20 (1.0)	8 (40.0)	12 (60.0)		9.9	4.1–23.7
No	2050 (99.0)	123 (6.0)	1927 (94.0)			
Down's syndrome				0.795		
Yes	1 (0.1)	0 (0.0)	1 (100.0)		–	
No	2069 (99.9)	131 (6.3)	1938 (93.7)		1	
Hospitalisation				<0.001		
Yes	236 (11.4)	40 (16.9)	196 (83.1)		3.0	2.2–4.0
No	1834 (88.6)	91 (5.0)	1743 (95.0)		1	
ICU admission				<0.001		
Yes	111 (5.4)	21 (18.9)	90 (81.1)		3.5	2.2–5.4
No	1958 (94.6)	110 (5.6)	1848 (94.4)		1	
Symptoms–survey end, median (range)	19 (12–23)	11 (7–15)	20 (14–24)	<0.001		
Symptoms–ICU admission, median (range)	6 (3–9.5)	5 (3–8)	7 (3–10)	0.19		
ICU admission–survey end, median (range)	125 (8–17)	6 (3–8)	13 (9–18)	<0.001		
Total	2070 (100.0)	131 (6.3)	1939 (93.7)			

RR, relative risk; 95% CI, 95% confidence interval.

Fortaleza-Ceará-Brazil, 2020.

Death occurred in 19.9% of the elderly people (*P* < 0.001), 56.6% in people with CVD (*P* < 0.001), 49.1% in people with diabetes (*P* < 0.001), 68.7% in people with neurological diseases (*P* < 0.001) and 52% in people with pneumopathies (*P* < 0.001). Mortality in 16.9% of hospitalised people (*P* < 0.001) and 18.9% in those admitted in ICU (*P* < 0.001) was also seen (Table 1).

The mortality risk was 3.7 times higher in the elderly (95% CI 3.2–4.2), 19.3 times higher in people with CVD (95% CI 14.7–25.2), 14.3 times higher in people with diabetes (95% CI 10.3–19.7), 32.6 times higher in people with neurological disease (95% CI 11.5–92.3) and 9.9 times higher in people with renal disease (95% CI 4.1–23.7). Such a risk was 3.0 times higher in hospitalised people (95% CI 2.2–4.0) and 3.5 times higher in people admitted to ICU (95% CI 2.2–5.4) (Table 1).

In the robust Poisson regression, time variables were not included due to missing values and T2 (time since the beginning of symptoms until death/end of the survey) was used only in the survival analysis. After adjustment, risk factors to death are: age range ≥60 years (IRR = 3.1; IC 95% 1.9–5.0; *P* < 0.001), neurologic disease (IRR = 3.7; 95% CI 1.8–7.9; *P* < 0.001) and pneumopathies (IRR = 2.0; 95% CI 1.1–3.9; *P* = 0.04). It is highlighted that people with CVD had 9.5 times higher risk of death (*P* < 0.001); however, attention should be given to its large confidence interval (95% CI 5.4–17.0) (Table 2).

**Table 2.** Robust Poisson regression model of the risk factors to death by COVID-19

	IRR	*P*-value	95% CI
**Age range (≥60 years)**	3.1	<0.001	1.9–5.0
Sex (male)	1.2	0.17	0.9–1.7
**Cardiovascular disease**	9.5	<0.001	5.4–17.0
Diabetes	1.5	0.08	1.0–2.3
Hematologic disease	0.7	0.66	0.2–3.0
**Neurologic disease**	3.7	<0.001	1.8–7.9
Obesity	3.5	0.13	0.7–18.0
**Pneumopathies**	2.0	0.04	1.1–3.9
Renal disease	0.6	0.12	0.3–1.2
Constant	0.01	<0.001	0.01–0.02

IRR, incidence rate ratio; 95% CI, 95% confidence interval. *R*^2^ = 34.09.

Fortaleza-Ceará-Brazil, 2020.

**Fig. 1.**
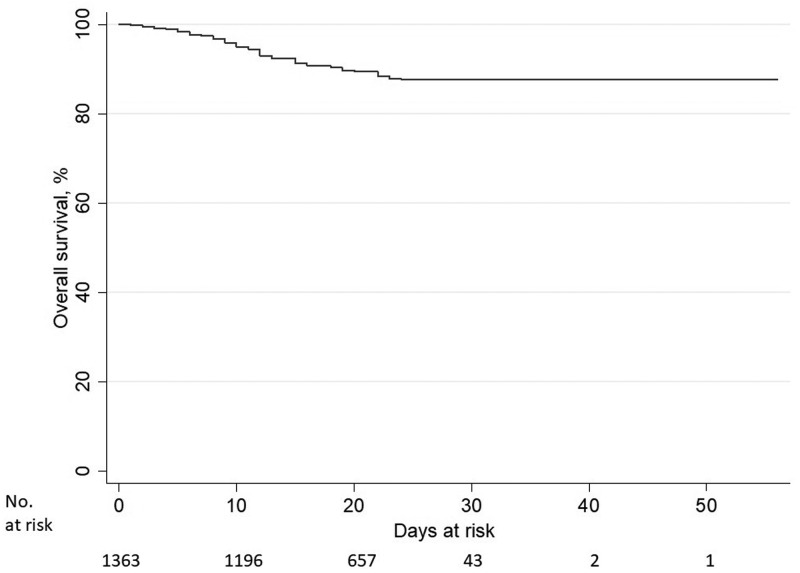
Kaplan–Meier survival function of people with COVID-19. Fortaleza-Ceará-Brazil, 2020.

Regarding survival analysis, it was considered the time since the beginning of symptoms until death/end of the survey (T2) as the study time variable. However, only 1363 out of 2070 confirmed cases had this record. Thus, 131 deaths by COVID-19 were analysed in 24 854 people-days at risk. When evaluating the Kaplan–Meier survival function, 99.9% of survival probability in the 1st day, 95.1% in the 10th and 89.7% in the 20th was observed. From the 24th day of the disease course, the survival rate has been around 87.7% (Fig. 1).

**Fig. 2.**
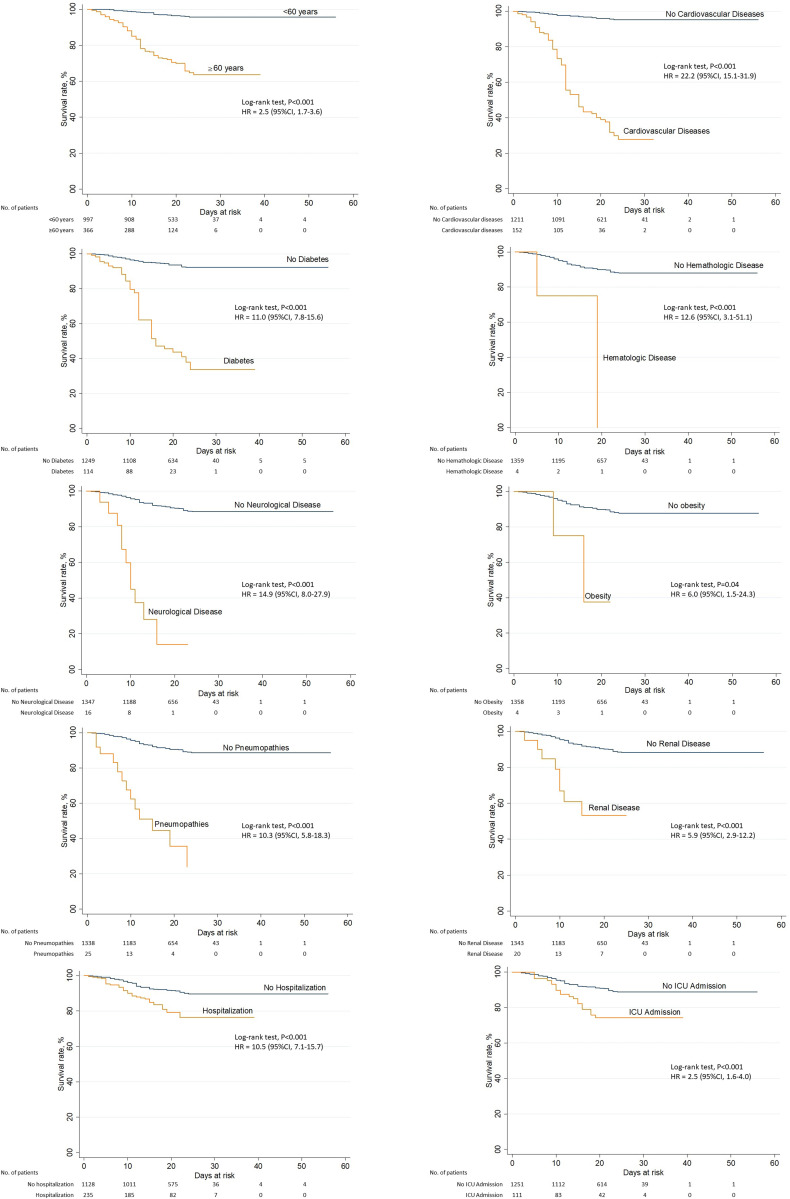
Survival in the presence of a characteristic associated with death in COVID-19 cases. Fortaleza-Ceará-Brazil, 2020.

By applying the log-rank test, differences in the survival functions were observed in the variables: age range (*P* < 0.001), CVD (*P* < 0.001), diabetes (*P* < 0.001), hematologic disease (*P* < 0.001), neurological disease (*P* < 0.001), obesity (*P* < 0.001), pneumopathies (*P* < 0.001), renal diseases (*P* < 0.001), hospitalisation (*P* < 0.001) and ICU admission (*P* < 0.001). Differences in these curves can be seen in Figure 2.

In the Cox regression model, all variables that presented differences in the survival curves were inserted, except hospitalisation and ICU admission due to the risk of bias. After adjustment, a risk of 3.6 higher to elderly people (95% CI 2.3–5.8; *P* < 0.001), 3.9 times in people with neurologic disease (95% CI 1.9–7.8; *P* < 0.001) and 2.6 times in people with pneumopathies (95% CI 1.4–4.7; *P* < 0.001) was identified. The highest risk of COVID-19 death was in people with CVD, with 8.9 times higher (*P* < 0.001), but with a large confidence interval (95% CI 5.4–14.5) (Table 3).

**Table 3.** Cox regression of the risk factors to death in COVID-19 cases

	Hazard ratio	*P*-value	95% CI
**Age range (elderly)**	3.6	<0.001	2.3–5.8
**Cardiovascular disease**	8.9	<0.001	5.4–14.5
Diabetes	1.3	0.21	0.8–2.1
Hematologic disease	3.6	0.09	0.8–16.1
**Neurologic disease**	3.9	<0.001	1.9–7.8
Obesity	1.7	0.51	0.4–7.7
**Pneumopathies**	2.6	<0.001	1.4–4.7
Renal disease	0.7	0.37	0.3–1.6

95% CI, 95% confidence interval.

Fortaleza-Ceará-Brazil, 2020.

## Discussion

In the Northeast of Brazil, Ceará has the highest incidence of infection among all states in Brazil [5] and 6.3% in the cumulative incidence of mortality, similar to countries with the highest number of cases, such as Iran, China and the USA [3]. In the State, ICU admission has occurred around the 6th day after the onset of symptoms, below 10.5 or 12 as observed in a study conducted in hospitals in Wuhan, China, showing moderate to severe symptoms when looking for services [6, 7]

Death happens shortly after (around the 19th day) than is seen in other regions of the world, whose median is 14 days (6–41) or 18.5 days (15–22) after the beginning of symptoms [6, 7]. This time may be due to vacancies not yet saturated in the local health system, allowing quick access to hospitals.

When comparing T3 (ICU admission until death/end of the survey), the 12.5 days of the present study were longer than in China, around 6.5 days [7]. This study observed that survival stabilises on the 24th day of the disease course. Thus, early identification and timely treatment of critical cases are crucial for decreasing the number of deaths and increasing survival.

The elderly and people with comorbidities (CVD, neurological disease, lung disease) had a higher risk of dying in both the Poisson and Cox models.

This study confirms the epidemiology of the disease. According to the Chinese Center for Disease Control, the mortality rate is largely influenced by the age of patients (>60 years), reaching 14.8% in those with >80 years [8]. In Italy, deaths occurred mainly in elderly men with multiple comorbidities. Similar to Ceará, these data remain limited and derived from the first month of documented cases of COVID-19 in Italy. As other patients currently infected may die shortly, this pattern of mortality may change [9].

Preliminary reports of 4226 patients with COVID-19 in the USA indicated higher mortality in people aged ≥85 years, especially those with some chronic disease [10]. The weaknesses of advanced age are related to the function of defence cells T and B, and to the excess production of type 2 cytokines, which can lead to a prolonged pro-inflammatory response, potentially leading to poor results [11]. This plus the high expression of angiotensin-converting enzyme genes in different parts of the body, such as heart and lungs, may increase death risk in this group.

Several studies have reported a higher risk of dying in the elderly and those with comorbidities, especially CVD [12–14], not differing from the findings of the current study. However, patients in most of these studies were already hospitalised. Despite this, this variable remained in the final Poisson regression model, being a risk factor for death, which seems to be similar to the rest of the world.

Regarding survival (where the time factor is associated), it was lower in the elderly with comorbidities, similar to what was observed in other studies; however, there is divergence regarding the probabilities over time, since international studies have shorter survival rates to a shorter time interval from the onset of symptoms of death [12, 15].

The main limitation of this study arises from the use of a secondary database, which limits the predictors to the existing data and hinders patient monitoring. Limitations are also related to the patient's answer or the health professional judgment with no further confirmatory test; for example, obesity was only subject to health professional interpretation, being considered only in advanced stages of it. Moreover, diabetes and hypertension, as silent diseases, may be subject only to the answer of those diagnosed with any of them. Missing values of time were also considered as potentially limited survival analysis. Even with its limitations, the results presented in this article are similar to the world literature. It can be a base of generalisation of the epidemiological aspects of COVID-19 and, therefore, define a profile of it for Brazil as a whole.

## Conclusion

Mortality by COVID-19 in Ceará is similar to countries with a large number of cases of the disease, although deaths occur later. The data presented show that the population seeks health services with the first symptoms of COVID-19, which facilitates early diagnosis and adequate treatment on time, to avoid the unfavourable prognosis of the disease. Besides, the elderly and people with comorbidities have a higher risk of death and shorter survival. By knowing the mortality of COVID-19 and the survival of the patients affected by the disease, it is possible to identify risk factors for the occurrence of death, allowing specialised care adopted for its prevention.

## Data Availability

The data that support the findings of this study are available from IntegraSUS website from the Ceará State Government (https://integrasus.saude.ce.gov.br/).
